# Research Advances in the Pathogenesis of Sepsis-Associated Encephalopathy

**DOI:** 10.3390/ijms27125390

**Published:** 2026-06-15

**Authors:** Haowen Tan, Wei Su, Zhendong Niu

**Affiliations:** 1 Department of Emergency Medicine, West China Hospital, Sichuan University, Chengdu 610041, China; tanhaowen0427@163.com; 2 West China School of Clinical Medicine, Sichuan University, Chengdu 610041, China; 3 Department of Science and Technology, West China Hospital, Sichuan University, Chengdu 610041, China; suwei0534816@163.com

**Keywords:** sepsis-associated encephalopathy, sepsis, blood–brain barrier, inflammatory response, cell-mediated immunity, pyroptosis, ferroptosis, autophagy, gut–brain axis, microglia

## Abstract

Sepsis-associated encephalopathy (SAE) is a frequent neurological complication of sepsis, driven by six interconnected pathophysiological components: (1) systemic inflammation-triggered neuroinflammatory cascades, initiated by systemic recognition of pathogen-associated molecular patterns (PAMPs) and damage-associated molecular patterns (DAMPs) and propagated by pro-inflammatory mediators; (2) central nervous system (CNS) immune cell-mediated neuroinflammation, wherein microglia, regulatory T cells, and neutrophils dynamically regulate inflammatory progression; (3) blood–brain barrier (BBB) disruption, progressing from functional disturbance to structural damage via tight junction degradation and immune infiltration; (4) multimodal programmed cell death, encompassing autophagy, apoptosis, pyroptosis, and ferroptosis driven by mitochondrial dysfunction; (5) neurotransmitter network imbalance, manifesting as cholinergic deficiency and glutamate excitotoxicity; and (6) gut–brain axis dysregulation, characterized by reduced microbiota-derived metabolites such as butyrate and indolepropionic acid. These components are organized along a core pathological axis comprising four sequential stages: neuroinflammatory storm (encompassing components 1 and 2) → BBB disruption and microcirculatory disturbances (component 3) → multimodal programmed cell death (component 4) → neurotransmitter imbalance (component 5), with the gut–brain axis (component 6) functioning as a bidirectional regulatory node that intersects and modulates all four stages. Mitochondrial dysfunction serves as the central converging node linking these pathological axes. Targeted interventions against neuroinflammation, immune cell modulation, BBB restoration, inhibition of aberrant cell death, neurotransmitter homeostasis, and gut microbiota remodeling hold therapeutic promise. Elucidating the crosstalk among these pathways will accelerate the clinical translation of precision therapies for SAE.

## 1. Introduction

Sepsis-associated encephalopathy (SAE) occurs in more than 50% of hospitalized sepsis patients and is associated with high mortality [1]. The core clinical features of SAE include fluctuating consciousness disorders, executive dysfunction, and neurological deficits [1]. Patients with characteristic consciousness fluctuations face a three-to fivefold higher risk of death, with in-hospital mortality reaching 45–60% in severe cases [2]. Although SAE has been directly linked to worse sepsis outcomes, the complex interactions within the neuro-immune-vascular unit remain poorly understood. The crosstalk among different pathological components has not been fully elucidated. Moreover, most current research focuses on basic mechanisms, with few translational targets available for clinical practice, hindering the development of precise treatment strategies for SAE.

**Figure 1:**
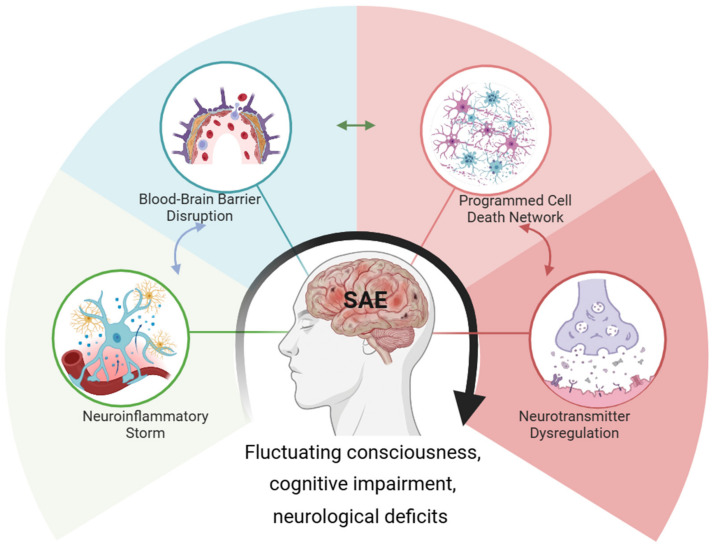
Core pathological axis and clinical manifestations of sepsis-associated encephalopathy (SAE). The central symptoms—fluctuating consciousness, cognitive impairment, and neurological deficits—are hypothesized to stem from possible interplay among four pathological axes, which are molecularly dissected into six pathological components in this review: (1) neuroinflammatory cascades and (2) immune cell-mediated regulation collectively constitute Axis 1, the neuroinflammatory storm; (3) BBB disruption and microcirculatory disturbances form Axis 2; (4) multimodal programmed cell death represents Axis 3; (5) neurotransmitter imbalance constitutes Axis 4. The gut–brain axis serves as a key regulatory node (dashed arrows) modulating all four axes. Bidirectional arrows denote plausible positive feedback loops suggested by accumulated evidence between adjacent axes: neuroinflammation exacerbates BBB disruption via cytokine-mediated tight junction degradation, while BBB leakage enables immune infiltration that may amplify neuroinflammation; BBB injury is associated with energy crisis and calcium overload that can trigger programmed cell death, whereas cell death releases DAMPs that further compromise barrier integrity; programmed cell death may disrupts neurotransmitter synthesis and synaptic function, and neurotransmitter imbalance (particularly cholinergic deficiency) likely impairs anti-inflammatory pathways, accelerating glial activation and cell death. Mitochondrial dysfunction serves is proposed as the central converging node linking all four axes. These proposed interactions are based on synthesizing evidence from diverse study designs; causal relationships remain to be validated by future dedicated investigations. Created in BioRender. Retrieved from https://BioRender.com/y7m9d3u on 7 May 2026.

Recent advances in interdisciplinary research paradigms and multi-omics technologies have accelerated mechanistic understanding. Breakthrough discoveries—such as glial cells revealed by single-cell sequencing, dynamic BBB leakage captured by live imaging, and cerebrospinal fluid neurotransmitter imbalances identified via metabolomics—have provided new dimensions for constructing a molecular map of SAE. This review systematically examines and synthesizes current evidence on six core pathological components organized along four sequential axes (Figure 1): (1) neuroinflammatory cascades (Section 3.1) and immune cell-mediated regulation (Section 3.2), collectively constituting the neuroinflammatory storm; (2) blood–brain barrier remodeling and microcirculatory disturbances (Section 3.3); (3) multimodal programmed cell death (Section 3.4); (4) neurotransmitter network imbalance (Section 3.5). The gut–brain axis (Section 3.6) serves as a key regulatory node modulating all four axes.

## 2. Materials and Methods

A comprehensive literature search was conducted in PubMed, Web of Science, and Embase to identify studies on sepsis-associated encephalopathy (SAE) pathogenesis and therapeutics published between January 2015 and March 2026. The search strategy combined MeSH terms and free-text keywords including “sepsis-associated encephalopathy,” “neuroinflammation,” “blood–brain barrier,” “programmed cell death” (autophagy, apoptosis, pyroptosis, ferroptosis), “gut–brain axis,” and related molecular targets. Boolean operators were used to connect disease terms with mechanism-specific keywords. Given the breadth of mechanisms covered, separate searches were conducted for each pathological component (neuroinflammation, BBB disruption, cell death, neurotransmitter imbalance, gut–brain axis). A representative PubMed search string for the cell death component was: (“sepsis-associated encephalopathy”[Title/Abstract] OR “SAE”[Title/Abstract]) AND (“programmed cell death”[Title/Abstract] OR “autophagy”[Title/Abstract] OR “apoptosis”[Title/Abstract] OR “pyroptosis”[Title/Abstract] OR “ferroptosis”[Title/Abstract]). Similar structures were applied to other components with appropriate mechanism-specific keywords substituted.

Inclusion criteria were: (1) original research articles, reviews, or clinical studies published in English; (2) studies investigating SAE pathogenesis involving neuroinflammatory cascades, BBB disruption, cell death pathways, or gut–brain axis dysregulation; and (3) for interventional studies, reporting clear mechanisms of action; review articles were included if they systematically summarized relevant evidence.

Exclusion criteria included case reports with fewer than 3 subjects, conference abstracts without full text, and non-peer-reviewed publications.

The literature selection followed a three-stage process. Initial screening of titles and abstracts identified 2500 records, of which 380 underwent full-text review. Ultimately, 116 references were included in the primary analysis, comprising preclinical original articles, clinical studies, and reviews; additional recent studies were incorporated during revision to ensure currency.

Data were extracted and synthesized according to four core pathological components consistent with the review’s framework: neuroinflammatory storms, BBB remodeling, neurotransmitter network imbalances, and gut–brain axis dysregulation. Intervention strategies were critically classified by clinical translation stage: clinically available drugs, preclinical candidates, and potential molecular targets. This narrative review employed a critical appraisal approach, prioritizing studies with robust experimental designs and validated methods while acknowledging limitations and interpreting conflicting findings with caution.

Notably, the proposed integrated pathological axis is established by synthesizing evidence from diverse study designs. Most of the included studies are observational or associative in nature, rather than being purposely designed to verify the causal relationships of this integrated model. The causal interactions among these pathological components remain to be validated by dedicated future studies.

## 3. Pathogenesis and Therapeutic Strategies

### 3.1. Neuroinflammatory Cascades and Targeted Therapies

The proposed core pathological mechanism of SAE is initiated by a systemic inflammatory storm. Recognition of PAMPs/DAMPs triggers innate immunity, leading to a cascade release of pro-inflammatory mediators such as IL-1β, IL-6, TNF-α. These circulating factors cross the BBB or enter the CNS via pathways like the vagus nerve, activating microglia and astrocytes and forming a self-amplifying neuroinflammation cycle [3].

Neuroinflammation appears to damage neuronal function in part by directly attacking mitochondria. TNF-α and IL-1β released by activated glial cells bind to death receptors on neuronal membranes, inducing persistent opening of the mitochondrial permeability transition pore (mPTP). This leads membrane potential collapse, ATP synthesis interruption, and cytochrome c leakage, thereby initiating apoptosis [4]. Simultaneously, NADPH oxidase (NOX2) and inducible nitric oxide synthase (iNOS) are strongly upregulated in M1-polarized microglia, resulting in burst production of superoxide anion (O_2_•^−^) and nitric oxide (NO). These combine to form peroxynitrite (ONOO^−^), a potent oxidant that directly damages mitochondrial electron transport chain complexes and induces mitochondrial DNA damage [5]. Inflammatory signaling also inhibits PINK1/Parkin-mediated mitophagy and disrupts PGC-1α-regulated mitochondrial biogenesis, leading to the accumulation of damaged mitochondria and widespread energy metabolism imbalance [6].

Parallel to and exacerbating neuroinflammation is severe cholinergic system dysfunction. In the septic environment, choline acetyltransferase (ChAT)-positive neurons in key brain regions such as the hippocampus are reduced in number and activity, leading to insufficient synthesis of the anti-inflammatory mediator acetylcholine (ACh) [7]. At the same time, acetylcholinesterase (AChE) activity is upregulated by inflammatory signals, accelerating ACh breakdown in the synaptic cleft. Consequently, the cholinergic anti-inflammatory pathway (CAP)—a core bridge between neural and immune systems—is impaired. Expression or downstream signaling (e.g., JAK2/STAT3) of its key receptor, the α7 nicotinic acetylcholine receptor (α7nAChR), is impaired in the inflammatory milieu, preventing ACh from effectively inhibiting nuclear factor kappa-light-chain-enhancer of activated B cells (NF-κB) nuclear translocation and pro-inflammatory factor transcription [8]. More fundamentally, sepsis-induced systemic disruption can reduce vagal efferent tone, decreasing ACh release at its source and causing widespread suppression of this physiological anti-inflammatory system [9].

Given these mechanisms, multidimensional intervention strategies are needed. Dexmedetomidine, beyond its sedative effects via α2 receptors, activates the neuronal mitochondrial deacetylase Sirt3, improving oxidative phosphorylation and reducing reactive oxygen species (ROS) production [10,11]. Enhancing mitophagy via the PINK1/Parkin pathway has also emerged as a potential strategy [3]. For the cholinergic system, selective α7nAChR agonists (PNU-282987) can more precisely activate the CAP and inhibit microglial activation [12]. Additionally, targeting the TLR4/NF-κB axis with guggulsterone (preclinical research) and selective mitochondrial antioxidant protection via high-concentration hydrogen inhalation are promising approaches [13,14]. Implementing these multi-dimensional strategies requires a deeper understanding of how different immune cells—from resident microglia to infiltrating T cells and neutrophils—dynamically regulate neuroinflammation. Notably, the central role of mitochondrial dysfunction as a converging node (Figure 1) becomes particularly evident when examining immune cell activation: microglial M1 polarization, T cell metabolic reprogramming, and neutrophil NETosis are all energy-intensive processes dependent on mitochondrial integrity. The following section details this immune cell-mediated regulation and its therapeutic targeting.

### 3.2. Immune Cell-Mediated Regulation of Neuroinflammation

**Figure 2:**
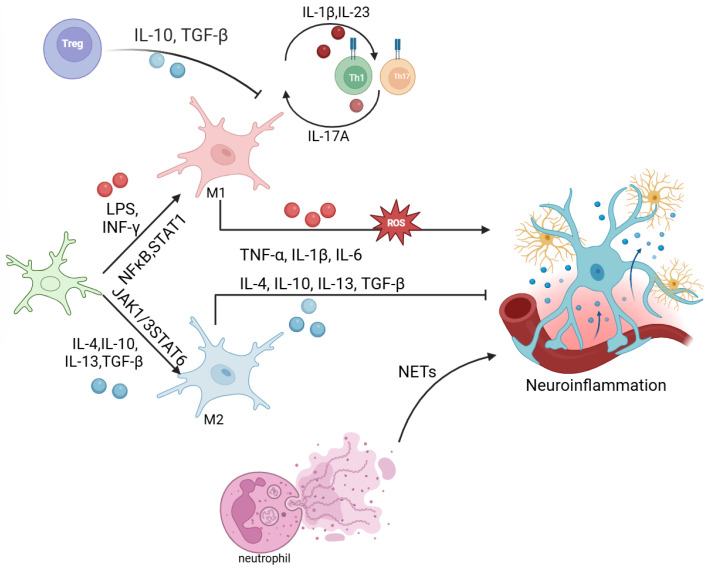
Neuroinflammation mediated by immune cells. Immune cell crosstalk in the regulation of neuroinflammation. Treg: Regulatory T cells; IL: Interleukin; TGF-β: Transforming growth factor beta; LPS: Lipopolysaccharide; INF-γ: Interferon gamma; NFKB: Nuclear factor kappa-light-chain-enhancer of activated B cells; STAT: Signal Transducer and Activator of Transcription; ROS: Reactive Oxygen Species; TNF-α: Tumor necrosis factor alpha; Th1: Type 1 T helper cell; Th17: Type 17 T helper cell; M1: M1 macrophage; M2: M2 macrophage; NETs: Neutrophil Extracellular Traps. Created in BioRender. Retrieved from https://BioRender.com/wfjuk3n on 7 May 2026.

While Section 3.1 focused on how peripheral inflammation triggers neuroinflammation and directly damages neurons via mitochondrial dysfunction, this section delves into the complex regulatory network of immune cells—both CNS-resident (microglia) and infiltrating (T cells, neutrophils, mast cells)—that determines the intensity and outcome of neuroinflammation. These cells drive the initiation and amplification of neuroinflammatory cascades, thereby promoting subsequent cell death and neurotransmitter imbalance (Figure 2).

#### 3.2.1. The Dynamic Regulatory Network of Microglia

Under physiological conditions, microglia surrounding brain microvessels help maintain BBB integrity by enhancing tight junction proteins [15]. During sepsis, these cells undergo dynamic functional transformation: early activation exerts protective effects via Foxc1-mediated IκBα/NF-κB signaling regulation [16]; however, sustained activation triggered by the RAGE ligand S100B exacerbates abnormal BBB permeability [17,18]. Infiltration of peripheral TH1/TH17 cells into the CNS leads to aberrant IL-17A secretion, which further promotes persistent microglial activation via a positive feedback loop [19]. Activated microglia secrete pro-inflammatory factors such as TNF-α, IL-6, and HMGB1. In addition to the mitochondrial damage induced by TNF-α, TNF-α also induces excitotoxic neuronal injury by causing abnormal glutamate secretion from glial cells and inhibiting astrocytic reuptake [20].

Intervention strategies targeting microglia are classified by clinical transformation stage: ① Preclinical research: CD137L neutralizing antibody TKS-1 inhibits M1 polarization [21]; α7nAChR agonist remimazolam activates the Nrf2/HO-1 pathway [22]; miR-25-3p inhibits IL-1β signaling via the TLR4/NLRP3 axis [23]; miR-494 maintains mitochondrial metabolic homeostasis [24]. HMGB1 inhibitors restore synaptic pruning balance [25,26]; MFGE8 enhances αVβ integrin-mediated efferocytosis [27,28]; the microglial depletion agent PLX5622 prevents synaptic engulfment [29]. ② Natural Compounds: β-patchoulene, β-elemene, and resveratrol glycoside exert anti-inflammatory effects by modulating pathways such as Sirt1/Nrf2 and RAC1/MLK3/p38 [30,31,32].

#### 3.2.2. Regulation of Neuroinflammation by Regulatory T Cells

Regulatory T cells (Tregs) maintain tissue microenvironmental balance by suppressing excessive immune responses and can migrate across the BBB into CNS. Activation of nicotinic acetylcholine receptors on their surface promotes clonal expansion within the hippocampus and modulates neuroinflammation via acetylcholine signaling [33,34]. In SAE, brain-infiltrating Tregs antagonize microglial inflammatory cascades by secreting factors like IL-10 and TGF-β, and suppress astrocytic IL-6 synthesis through the amphiregulin (AREG)-EGFR signaling axis [35,36]. Caspase-1-mediated GSDMD cleavage, indicating activation of the pyroptosis pathway, has been observed in peripheral blood immune cells of sepsis patients, suggesting the involvement of programmed cell death mechanisms in disease progression [37].

#### 3.2.3. Synergistic Effects of Neutrophils and T Cells

In sepsis models, elevated levels of IL-1β and IL-6 are accompanied by neutrophils and T cells infiltration [38]. Among these, Treg/Th2 cells can improve SAE-associated psychiatric disorders by alleviating chronic-phase inflammation. The γδ T cell subset exacerbates microglial activation via IL-17A signaling, and intervention with anti-γδTCR/anti-IL-17A significantly ameliorates anxiety-like behavior [39]. Neutrophils and their Neutrophil Extracellular Traps (NETs) mediate BBB disruption, neuronal death, and microglial activation in the hippocampus. Targeted interventions (anti-Gr-1 antibody/DNase I) or gene knockout (GSDMD/PD-L1) can significantly improve the pathological progression. Mechanistically, phosphorylated STAT3 (p-Y705) in neutrophils forms a complex with PD-L1, driving nuclear translocation and upregulating GSDMD expression to promote NETosis [40]. Kynurenic acid (KYNA) exerts neuroprotective effects by inhibiting NET formation, maintaining BBB integrity, and improving mitochondrial function [41]. Clinical studies confirm that cerebrospinal fluid NGAL can serve as a diagnostic biomarker for SAE [42].

Mast cells exacerbate BBB injury and cognitive impairment through the histamine/H1R-TLR2/4-MAPK axis, enhancing the inflammatory response in BMECs. The cascade reaction between myeloid cells and vascular endothelium induces microthrombus formation and perfusion disturbances, forming a central regulatory network of the vascular-immune-neural axis [43].

### 3.3. Dynamic Remodeling of the Blood–Brain Barrier and Intervention Strategies

The BBB is a sophisticated structure composed of brain microvascular endothelial cells (BMECs) with their tight junctions (TJ), basement membrane, astrocytic end-feet, and pericytes, serving as the critical defensive line for CNS homeostasis [44]. BBB remodeling in SAE progresses through distinct stages with differential molecular signatures and therapeutic windows (Table 1). Understanding this temporal sequence is critical for timing interventions.

In SAE, the BBB undergoes a dynamic remodeling from functional disturbance to structural disruption. The systemic inflammatory storm initiates this process. Circulating TNF-α and IL-1β activate the NF-κB and ROCK signaling pathways within endothelial cells, downregulating tight junction proteins (Occludin, Claudin-5, and ZO-1) and enhancing matrix metalloproteinases (MMP-2/9) activity, which cleaves TJ proteins and promotes their endocytosis, leading to early, reversible increases in permeability [44]. Concurrently, the endothelial glycocalyx is sheds under inflammation and abnormal shear stress, shed components (e.g., syndecan-1) act as DAMPs to amplify inflammation, directly weakening the first physical and charge barrier against macromolecule extravasation [45,46].

BBB integrity highly depends on normal endothelial mitochondrial function. In the inflammatory environment, BMEC mitochondria are also attacked. Uncoupling of oxidative phosphorylation reduces ATP generation, while maintaining TJ structure and supporting active transport are highly energy-consuming processes; this energy crisis directly impairs barrier function [47]. Simultaneously, ROS produced within endothelial cells can oxidatively damage TJ proteins and activate the NLRP3 inflammasome, leading to caspase-1 activation and IL-1β maturation, exacerbating local disruption in an autocrine manner [48]. Mitochondrial dysfunction also causes loss of calcium ion buffering capacity, leading to intracellular calcium overload. This in turn activates calcium-dependent proteases to cleave TJ proteins and induces endothelial cell apoptosis, driving the progression of the BBB from functional leakage to structural damage [49].

BBB injury is associated with opening the gateway for peripheral immune cell infiltration. Activated endothelial cells highly express adhesion molecules such as E-selectin, P-selectin, ICAM-1, and VCAM-1, which interact with neutrophils and monocytes, mediating their rolling, firm adhesion, and transendothelial migration. Infiltrating immune cells release toxic substances and form local inflammatory foci, establishing a vicious cycle [50].

Intervention strategies targeting BBB remodeling include: strengthening tight junctions—dihydroartemisinin (DHA) stabilizes TJ protein expression by inhibiting SNAI1 [51]; mirtazapine and cabergoline also show barrier-fortifying effects [52,53]. For inhibiting immune cell adhesion, citrate-coated gold nanoparticles (cit-AuNP) effectively inhibit ICAM-1-mediated neutrophil adhesion. Multifunctional drugs such as rosuvastatin, memantine, and metformin protect endothelial cells and maintain BBB integrity through anti-inflammatory, antioxidant, and anti-excitotoxic pathways [54,55,56]. Furthermore, treatments targeting impaired meningeal lymphatic drainage (e.g., promoting VEGF-C expression) help accelerate the clearance of harmful substances from the brain, creating a favorable microenvironment for BBB repair [57].

### 3.4. MultiModal Programmed Cell Death Interaction Mechanisms

The neuroinflammatory environment likely serves as a key driver of multimodal programmed cell death (autophagy, apoptosis, pyroptosis, ferroptosis). These death pathways are interconnected and mutually regulated, collectively constituting the characteristic pathological alterations in SAE, with mitochondrial dysfunction as the common regulatory axis. For instance, excessive pyroptosis may consume cellular ATP and trigger secondary apoptosis, while defective autophagy can concurrently aggravate neuronal apoptosis and ferroptosis. Consistent with these interactive effects, GSDMD deficiency simultaneously suppresses pyroptosis and nuclear autophagy [58], while GSDMD/Drp1-mediated mitochondrial fission contributes to synaptic damage [59]. Dysregulated mitochondrial quality control, including VDAC1-related mechanisms and impaired PINK1/Parkin-mediated mitophagy, further links autophagy dysfunction to neuronal injury [60,61].

#### 3.4.1. Dynamic Regulation and Therapeutic Interventions of Autophagy and Apoptosis

In SAE progression, the interplay between autophagy and apoptosis is a key pathological mechanism. CXCR5 reduces hippocampal autophagy activity by inhibiting the p38 MAPK/NF-κB/STAT3 pathway; targeted intervention can restore autophagic flux and ameliorate cognitive impairment [58]. Conversely, silencing VDAC1 enhances mitochondrial quality control by activating the PINK1/Parkin pathway, synergistically suppressing aberrant autophagy and promoting mitophagy [62]. IRGM1 inhibits neuronal apoptosis and activates protective autophagy by regulating p38 MAPK phosphorylation [60], while sestrin 2 exerts therapeutic effects by enhancing autophagy via the ULK1 pathway [63]. Pharmacological interventions show that dexamethasone has a dual dose-dependent effect: low doses regulate autophagy via mTOR inhibition, whereas high doses activate apoptotic pathways [61].

Mitochondrial regulatory networks indicate that Nogo-A induces mitophagy and apoptosis via the ROS-p-SHP2 axis [64], while Omi/HtrA2 promotes apoptosis through dual mechanisms: inhibiting XIAP and inducing mitochondrial membrane potential depolarization [65]. Blocking the MD-2-mediated apoptosis-necroptosis signaling interaction significantly improves neuronal survival [66]. Intervention strategies classified by clinical transformation stage: ① Clinically available drugs: Melatonin induces M2 microglial polarization through AMPKα2 activation [67]. ② Preclinical research: Emodin simultaneously inhibits apoptosis and activates autophagy via the BDNF/TrkB signaling pathway [68]; umbilical cord mesenchymal stem cells mitigate inflammation and apoptosis via the PI3K/AKT pathway [69]; fisetin inhibits IL-1β secretion via the mitophagy-NLRP3 axis [70]; NMN alleviates oxidative damage through the NAD+/SIRT1 pathway [71]; Lrg1 silencing reduces neuronal apoptosis by suppressing the TGFβ1/SMAD signaling pathway [72]; ③ Non-pharmacological interventions and potential targets: Environmental enrichment (EE) exerts protective effects by enhancing antioxidant, anti-inflammatory, and anti-apoptotic responses [73]; FGR gene silencing regulates mitochondrial homeostasis via the Sirt1/PGC-1α pathway [74].

#### 3.4.2. Molecular Regulatory Networks and Targeted Interventions of Pyroptosis

**Figure 3:**
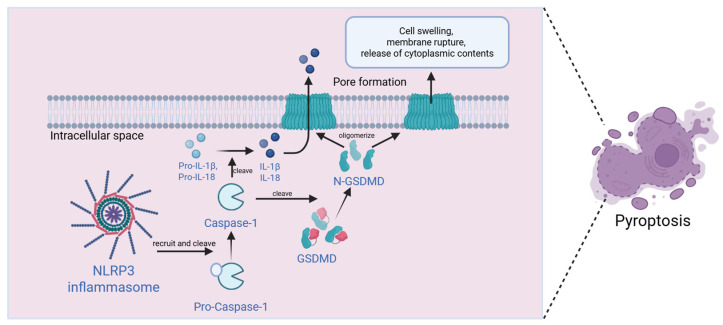
Canonical pyroptosis pathway mediated by the NLRP3 inflammasome. Pathogen-associated molecular patterns (PAMPs) or damage-associated molecular patterns (DAMPs) activate the NLRP3 inflammasome, which recruits and cleaves pro-caspase-1 into active caspase-1. Active caspase-1 executes two critical functions: (1) it cleaves gasdermin D (GSDMD) into its N-terminal fragment (N-GSDMD), which oligomerizes and forms pores in the plasma membrane, leading to cell swelling, membrane rupture, and the release of cytoplasmic contents (including LDH and HMGB1); (2) it cleaves pro-IL-1β and pro-IL-18 into their mature forms, which are released through GSDMD pores to amplify local and systemic inflammatory responses. This positive feedback loop perpetuates neuroinflammation and exacerbates blood–brain barrier disruption in SAE. Abbreviations: IL, interleukin; GSDMD, gasdermin D; N-GSDMD, N-terminal fragment of GSDMD; NLRP3, NLR family pyrin domain containing 3; LDH, lactate dehydrogenase; HMGB1, high mobility group box 1. Created in BioRender. Retrieved from https://BioRender.com/r8y030v on 7 May 2026.

In SAE, NLRP3 inflammasome-mediated pyroptosis is a central pathological mechanism. The NLRP3/caspase-1/GSDMD signaling axis is abnormally activated in the hippocampus of cecal ligation and puncture (CLP) models, accompanied by elevated pyroptosis markers and pro-inflammatory factors, leading to cognitive impairment [75]. Genetic knockout experiments demonstrate that GSDMD deficiency simultaneously inhibits pyroptosis and autophagy [58], while dynamin-related protein 1 (DRP1) inhibitors significantly improve pathological phenotypes by reversing mitochondrial dysfunction [59] (Figure 3).

Molecular mechanisms include:

(1) Ion channel regulation: The P2X7 receptor activates cortical pyroptosis pathways via ERK1/2 phosphorylation [76]; pannexin-1 regulates the autophagy-pyroptosis balance through AMPK signaling [77].

(2) Epigenetic regulation: TRIM45 aggravates microglial pyroptosis by promoting K63-linked polyubiquitination ubiquitination and stabilization of Atg5, thereby disrupting autophagic flux and activating the NLRP3 inflammasome [78]; Maf1 inhibits NF-κB/p65-driven inflammasome assembly [79].

Intervention strategies classified by clinical transformation stage: ① Preclinical research: Ethyl pyruvate inhibits NLRP3 [80]; TXNIP knockdown protects against sepsis-induced brain injury and cognitive decline by suppressing oxidative stress and neuroinflammation [81]; mesenchymal stem cell-derived exosomes deliver miR-140-3p to inhibit HMGB1-mediated pyroptosis [82]; hydrogen-rich saline blocks inflammasome activation via the Nrf2/autophagy pathway [83,84]; echinomycin improves mitochondrial structure and reduces inflammatory factors via the HIF-1α/BNIP3L axis [85]; ② Natural compounds (preclinical): Puerarin enhances BBB stability by blocking GSDMD cleavage; myricitrin alleviates neuroinflammation by modulating the Bax/Bcl-2 balance [86]; malvidin dually inhibits pyroptosis and apoptosis via the AMPKα/UCP2 axis [87].

#### 3.4.3. Molecular Regulatory Axes and Synergistic Interventions of Ferroptosis

**Figure 4:**
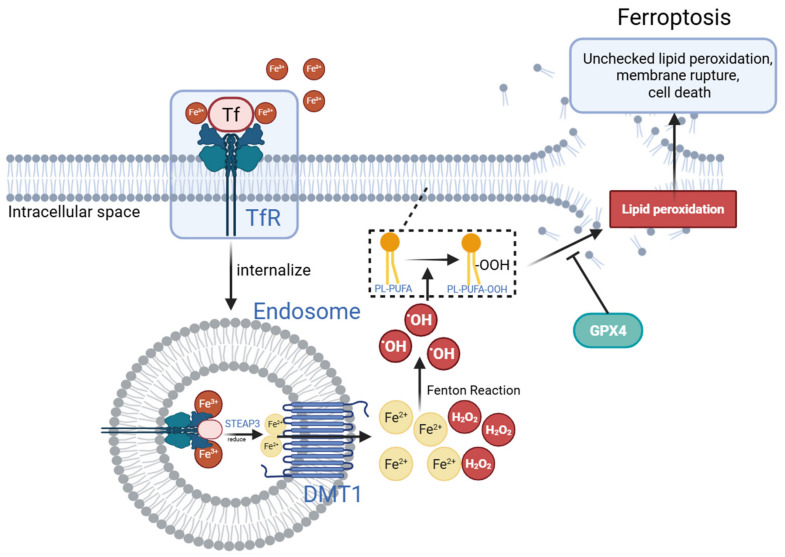
Canonical ferroptosis pathway involving iron metabolism and lipid peroxidation. The ferroptosis cascade proceeds through three sequential stages: (1) Iron accumulation: Transferrin (Tf) binds ferric iron (Fe^3+^) and delivers it to the transferrin receptor (TfR); the complex is internalized into endosomes, where STEAP3 reduces Fe^3+^ to ferrous iron (Fe^2+^), which is then exported to the cytosol via DMT1. (2) Lipid peroxidation: Cytosolic Fe^2+^ drives the Fenton reaction with hydrogen peroxide (H_2_O_2_) to generate highly reactive hydroxyl radicals (HO•), which attack polyunsaturated fatty acids (PUFAs) within membrane phospholipids (PL-PUFA), generating toxic lipid hydroperoxides (PL-PUFA-OOH). (3) Failed antioxidant defense and membrane rupture: Under normal conditions, glutathione peroxidase 4 (GPX4) converts PL-PUFA-OOH to non-toxic alcohols (PL-PUFA-OH), thereby inhibiting ferroptosis. In SAE, GPX4 activity is suppressed due to glutathione depletion and SLC7A11/xCT downregulation, leading to unchecked lipid peroxidation, membrane rupture, and cell death. Abbreviations: Tf, transferrin; TfR, transferrin receptor; Fe^2+^, ferrous iron; Fe^3+^, ferric iron; DMT1, divalent metal transporter 1; HO•, hydroxyl radical; PL, phospholipid; PUFA, polyunsaturated fatty acid; GPX4, glutathione peroxidase 4; STEAP3, six-transmembrane epithelial antigen of prostate 3. Created in BioRender. Retrieved from https://BioRender.com/926q8g1 on 7 May 2026.

Ferroptosis involves glutathione antioxidant axis dysfunction, iron metabolism disorders, and uncontrolled lipid peroxidation due to GPX4 activity inhibition. Clinical data show that SAE patients exhibit a 2.3-fold and 1.8-fold increase in peripheral blood S100β and GFAP levels, respectively, positively correlating with MDA accumulation (r = 0.72), alongside a 40% decrease in SOD activity, indicating oxidative/antioxidant system imbalance and mitochondrial cristae disruption [88] (Figure 4).

Multi-omics analyses reveal key ferroptosis regulatory networks in SAE: the hippocampal PEBP-1/15-LOX/ACSL4 axis is significantly activated, while GPX4 and xCT transporter (SLC7A11) expression is suppressed, forming a lipid peroxidation feedback loop [88]. Interventions include: (1) acetaminophen (APAP) restores the GPX4 pathway to inhibit ferroptosis [89]; (2) MAR1 reshapes the SLC7A11/GPX4 signaling pathway to suppress microglial activation [90]; (3) Exosomal NEAT1 exacerbates ferroptosis via the miR-9-5p/TFRC-GOT1 axis [91].

Pharmacological synergy studies demonstrate that propofol combined with dexmedetomidine achieves multi-target intervention by regulating the Fpn1 node, reducing FTH1 expression by 58% and MDA levels by 41%, while reversing iron transporter abnormalities [92]. Notably, ferroptosis regulation exhibits cell-context dependency: the EBF1-CSRP2 axis suppresses ferroptosis via SLC7A11/GPX4 modulation in leukemia [93], contrasting with its neurodestructive role in SAE. These findings provide systematic evidence for ferroptosis-targeted therapies in SAE, from molecular mechanisms to clinical translation.

### 3.5. Neurotransmitter Network Imbalance

#### 3.5.1. Core Mechanisms of Neurotransmitter Homeostasis Imbalance

Neurotransmitter homeostasis imbalance in SAE is associated with immune inflammation and cell death, involving metabolic disturbances of dopamine and glutamate and the synaptic plasticity damage they mediate [38]. Peripheral inflammation triggers central cytokine release via vagus nerve-mediated signal transduction, forming a vicious neuro-immune cycle [94]. Cholinergic system dysfunction is manifested by reduced ChAT-positive neurons and abnormal AChE activity, leading to dysregulated microglial activation thresholds and accelerated transformation towards neurotoxic phenotypes [94]. Functional imaging shows abnormally increased amplitude of low-frequency fluctuation (ALFF) values in the hippocampus, and the glutamate+glutamine (Glx)/creatine (Cr) ratio positively correlates with cognitive impairment, providing objective biomarkers for clinical assessment [95].

Molecular mechanism studies indicate that abnormal glutamatergic system activation induces excitotoxicity in the hippocampal-prefrontal cortical circuit via the N-methyl-D-aspartate receptor (NMDAR)/α-amino-3-hydroxy-5-methyl-4-isoxazolepropionic acid receptor (AMPAR)-cAMP response element-binding protein (CREB)-brain-derived neurotrophic factor (BDNF) axis, and specific receptor antagonists can reverse related molecular abnormalities [96,97]. LPS-induced abnormalities in septal-hippocampal cholinergic projections can be restored through circuit reprogramming to recover synaptic plasticity [98]. In epigenetic regulation, the Neat1/Hbb axis impairs dendritic spine density by downregulating postsynaptic density protein-95 (PSD-95) expression, while MFHAS1-siRNA intervention can significantly reduce Aβ pathological burden and repair dendritic morphology [99,100].

#### 3.5.2. Intervention Strategies Targeting Neurotransmitter Systems

Intervention strategies classified by clinical transformation stage: ① Clinically available drugs: N-acetylcysteine (NAC) protects the BBB through dual mechanisms of regulating glutamate metabolism and exerting antioxidant effects [101]; oxytocin improves cognition by reconstructing excitatory/inhibitory synaptic balance [102]; fluoxetine alleviates prefrontal dysfunction by restoring serotonergic (5-HT) signaling [103]; ② Preclinical research: Vagus nerve stimulation exerts anti-inflammatory effects by inhibiting endothelial activation cascades while regulating glial cells to maintain neuroprotective homeostasis.

### 3.6. Gut Microbiota-Gut–Brain Axis in SAE: Metabolic Regulation and Intervention Strategies

The gut–brain axis is proposed to mediate bidirectional communication between the gut and the CNS via immune-neuro-endocrine networks, and its dysfunction has been associated with SAE pathology. In SAE model mice, the abundance of short-chain fatty acid (SCFA)-producing bacteria (e.g., Bacteroides, Bifidobacterium) decreases by 62–75%, accompanied by a 54–68% reduction in acetate and propionate concentrations. Exogenous SCFA intervention can dose-dependently inhibit peripheral pro-inflammatory factor release and exert neuroprotection by upregulating tight junction proteins and blocking microglial JNK/NF-κB activation [104,105].

Molecular mechanisms involve: (1) Butyrate activates the GPR109A/Nrf2/HO-1 pathway [106]; (2) Indolepropionic acid (IPA) inhibits the NLRP3 inflammasome and IL-1β secretion [107]; (3) NU9056 upregulaes SCFA levels to antagonize inflammasome activity [108].

Microbiota remodeling strategies show that fecal microbiota transplantation (FMT) improves neurotransmitter balance by modulating the IBA-1 axis, inhibits M1 polarization and IL-1β cycling in mesenteric lymph node macrophages, and blocks gut epithelial exosome-mediated neuroinflammatory cascades [109]. Phosphatidylserine (PS) significantly downregulates hippocampal pro-inflammatory gene transcription by modulating TBK1/NLRP3 signaling [110]. Clinical data confirm abnormal ammonia metabolism in SAE patients. Mechanistic studies indicate the microbiota-gut–brain axis drives functional imbalance by inducing pathological overexpression of AQP4 in astrocytes [111,112].

Metabolic interventions: Molecular hydrogen improves metabolic-inflammatory imbalance via microbiota modulation [113]; propionate supplements exert protective effects by maintaining BBB integrity [114]. These findings systematically reveal the pivotal role of gut microbiota metabolic reprogramming in SAE, providing a theoretical basis for multi-dimensional interventions targeting the gut–brain axis.

Table 2 summarizes the key molecular mediators, upstream triggers, and downstream effects across all six pathological components discussed above, highlighting the central convergence of mitochondrial dysfunction.

## 4. Integrated Discussion and Critical Appraisal

### 4.1. Synthesis of the Core Pathological Axis

This review integrates current evidence on SAE into a unified framework comprising six molecular components organized along four sequential pathological axes: neuroinflammatory storm (encompassing neuroinflammatory cascades and immune cell-mediated regulation) → BBB disruption and microcirculatory disturbances → multimodal programmed cell death → neurotransmitter imbalance, with the gut–brain axis serving as a bidirectional regulatory node modulating all four axes. Mitochondrial dysfunction functions as the central converging node linking these pathological components.

A critical innovation of this framework is its explicit classification of intervention strategies by clinical translation stage—clinically available drugs, preclinical candidates, and potential molecular targets—thereby distinguishing this review from prior mechanistic descriptions and providing practical guidance for precision therapeutics. The mutual amplification between neuroinflammation and BBB damage emerges as a key trigger for SAE progression, while gut–brain axis dysregulation offers a non-invasive intervention entry point.

However, the translational trajectory from mechanistic understanding to clinical application remains uneven across components. Table 3 synthesizes the evidence base, key findings, and translational barriers for each pathological axis, revealing systematic gaps that underlie the limited clinical progress to date.

Several cross-cutting challenges emerge from this analysis. First, BBB permeability affects nearly all CNS-targeted agents, necessitating either high systemic doses with attendant toxicity or development of specialized delivery systems. Second, therapeutic timing represents a critical disconnect—preclinical studies universally administer interventions prophylactically or within hours of insult, whereas clinical SAE is typically recognized after established pathology, potentially beyond the window for mechanism-based intervention. Third, biomarker-guided stratification is absent, precluding identification of patients most likely to benefit from specific targets (e.g., NLRP3 inhibitors for inflammasome-high phenotypes, ferroptosis inhibitors for lipid peroxidation-dominant subgroups). Fourth, ICU-specific pharmacological constraints (hemodynamic instability, organ dysfunction, polypharmacy) complicate dosing and monitoring of investigational agents. These barriers highlight the necessity of iterative feedback between preclinical model refinement and early-phase clinical trial design, with particular attention to pharmacokinetic bridging studies and biomarker validation.

### 4.2. Critical Appraisal of Evidence and Unresolved Controversies

Beyond the translational barriers summarized in Table 3, several areas of conflicting evidence, methodological limitations, and unresolved controversies warrant deeper discussion to guide future research priorities.

#### 4.2.1. Conflicting Experimental Findings on Cell Death Interplay

The relationship between pyroptosis and autophagy remains contentious. GSDMD deficiency simultaneously suppresses both pyroptosis and autophagy in CLP models [58], suggesting pathway coupling; however, autophagy activation can precede and protect against pyroptosis by clearing damaged mitochondria [70,77]. Whether these pathways act synergistically to amplify injury or represent compensatory responses likely depends on injury severity, cell type, and temporal stage. Similarly, GPX4 inhibition triggers ferroptosis in some contexts while other studies implicate alternative death modalities [88,89] raising questions about context-dependent death modality preferences.

#### 4.2.2. Reproducibility Concerns and Model Heterogeneity

Reproducibility across SAE models appears to be suboptimal in existing published evidence. LPS injection produces rapid, transient neuroinflammation, whereas CLP induces polymicrobial sepsis with more sustained CNS pathology [3,75]. These models tend to yield divergent cytokine profiles and microglial activation kinetics. Furthermore, species differences in microglial density, BBB tight junction composition, and cholinergic system organization between rodents and humans likely restrict direct extrapolation of preclinical findings to human patients [15,16,18].

#### 4.2.3. Heterogeneity of SAE Definitions and Diagnostic Criteria

SAE lacks standardized diagnostic criteria, with studies employing heterogeneous definitions including Glasgow Coma Scale score, CAM-ICU positivity, EEG abnormalities, or composite scores [1,2]. This heterogeneity may introduce introduces selection bias and could hamper reliable biomarker validation. GCS-based definitions capture advanced encephalopathy but may miss subtle cognitive deficits, whereas EEG criteria require specialized expertise [1]. The absence of a unified SAE classification potentially hinders stratified therapy development.

#### 4.2.4. Limitations of Animal Models

Current animal models fail to fully replicate human SAE complexity. Most models use young, healthy mice, whereas human sepsis predominantly affects elderly patients with comorbidities that alter immune responses and BBB vulnerability [1,2]. Additionally, the absence of mechanical ventilation, sedation, and polypharmacy in animal models—standard in ICU settings—limits assessment of iatrogenic contributions to encephalopathy.

#### 4.2.5. Challenges in Clinical Translation

Multiple unresolved translational challenges largely hinder the clinical transformation of SAE targeted therapies. First, BBB permeability: many promising preclinical targets tend to exhibit poor CNS penetration, requiring doses that may risk peripheral toxicity [12,13,22]. Second, timing of intervention: most preclinical studies administer therapies prophylactically or early post-induction, whereas clinical SAE diagnosis often occurs after established pathology [3,75]. Third, biomarker availability: while NGAL, S100β, and GFAP appear to show diagnostic promise [42,88], none are validated for real-time monitoring. Fourth, multi-target complexity: proposed combination therapies face challenges in optimizing dosing schedules and demonstrating superiority over single-agent approaches in heterogeneous populations.

### 4.3. Concluding Remarks

SAE is a complex neuroinflammatory disease driven by the synergy of multiple interconnected components. We acknowledge that the proposed pathological axis serves only as a preliminary working framework based on synthesizing predominantly associative evidence from heterogeneous study designs and animal models. Critical gaps remain: the temporal dynamics of axis component interactions, the relative contributions of cell death modalities in human SAE, and the feasibility of multi-target combination therapies in complex ICU populations. Addressing these gaps through prospective, multi-center studies with standardized SAE definitions and integrated biomarker platforms will be crucial for validating this integrated pathological model.

Multi-target synergistic intervention targeting the core pathological axis and key regulatory nodes—particularly the gut–brain axis and mitochondrial function—may represent a promising direction for clinical translation. This integrated framework provides a systematic theoretical basis for the precise clinical management of SAE, while explicitly highlighting the limitations that need to be comprehensively addressed to fully unlock its translational potential.

## 5. Future Perspectives

Although significant progress has been made in elucidating pathophysiology, translation from basic research to clinical practice remains limited, particularly in the lack of precise biomarkers, personalized intervention strategies, and mature clinical trial evidence. Based on the core pathological axis and regulatory nodes summarized in this review, future research should focus on the following specific directions with clear translational paths:

(1) Deciphering the spatiotemporal dynamics of neuroimmune crosstalk: Investigate dynamic changes in microglial polarization, T-cell subset migration, and inflammatory mediator release across different brain regions (hippocampus, prefrontal cortex) and disease stages (early, middle, late). For example, combining single-cell sequencing to track microglial state transitions with live imaging to visualize dynamic BBB leakage in SAE models would enable precise mapping of neuroimmune interactions in real time. Clarify key time windows and spatial distribution rules of neuroimmune interaction to establish a precise intervention target map and avoid blind intervention.
(2) Promoting the clinical translation of SAE biomarkers: Further validate the diagnostic and prognostic value of candidate biomarkers (NGAL, S100β) in large-scale clinical cohorts. Optimize detection methods (e.g., combined cerebrospinal fluid and peripheral blood testing) and establish a combined biomarker evaluation system to enable early diagnosis, severity assessment, and prognostic prediction, laying a foundation for timely intervention.
(3) Accelerating development of gut–brain axis-targeted microecological preparations: Based on the regulatory role of SCFA-producing bacteria and metabolites (butyrate, indolepropionic acid) in SAE, develop personalized probiotic preparations, prebiotics, or FMT protocols targeting gut microbiota remodeling. Verify their safety and efficacy in preclinical studies and promote transition to clinical trials, providing new non-invasive intervention strategies for SAE.
(4) Optimizing the design of multi-target combination therapy: Based on the core pathological axis, design rational combined medication schemes, such as “NLRP3 inflammasome inhibitors (pyroptosis inhibition) + ZO-1 agonists (BBB repair) + mitochondrial protectants (energy metabolism improvement).” Clarify dosage, administration route, and combined efficacy in clinical trials, avoid single-target intervention limitations, and promote translation of multi-target synergistic therapy into routine clinical practice.
(5) Integrating AI-driven structural biology: AlphaFold-enabled protein structure prediction may accelerate rational design of BBB-penetrant agents targeting the core pathological axis [116].

## Figures and Tables

**Table 1:** Stages of blood–brain barrier remodeling in SAE: from functional disturbance to structural disruption.

Stage	Characteristics	Key Molecular Changes	Reversibility	Therapeutic Window
Stage I: Functional disturbance	Increased paracellular permeability; intact basement membrane; no immune cell infiltration	TJ protein internalization (Occludin ↓, Claudin-5 ↓, ZO-1 ↓); MMP-2/9 activation; ROCK signaling ↑; NF-κB nuclear translocation	Reversible	Optimal: DHA, mirtazapine, cabergoline
Stage II: Structural disruption	Basement membrane degradation; astrocytic end-feet detachment; pericyte loss; immune cell infiltration Partially reversible Limited: anti-inflammatory, antioxidant, lymphatic drainage promotion	TJ protein cleavage/degradation; glycocalyx shedding (syndecan-1 release); adhesion molecules ↑ (ICAM-1, VCAM-1, E-selectin); mitochondrial dysfunction (ATP ↓, Ca^2+^ overload)	Partially reversible	Limited: anti-inflammatory, antioxidant, lymphatic drainage promotion
Stage III: Irreversible damage	Microvascular necrosis; glial scar formation; persistent immune cell infiltration; microthrombosis	Caspase-3 activation; endothelial apoptosis; thrombin deposition; TGF-β ↑, collagen deposition	Irreversible	Palliative: neuroprotection, rehabilitation

Note: TJ, tight junction; MMP, matrix metalloproteinase; ROCK, Rho-associated protein kinase; ICAM-1, intercellular adhesion molecule-1; VCAM-1, vascular cell adhesion molecule-1; TGF-β, transforming growth factor-beta. Arrow direction indicates upregulation (↑) or downregulation (↓).

**Table 2:** Key pathological pathways and molecular mediators in SAE.

Pathological Axis	Key Molecular Mediators Upstream Triggers Downstream Effects Representative References	Key Molecular Changes	Reversibility	Therapeutic Window
Neuroinflammatory cascade	IL-1β, IL-6, TNF-α, HMGB1	PAMPs/DAMPs (LPS, S100B), TLR4/NF-κB activation	Microglial M1 polarization, astrocyte activation, mitochondrial ROS burst	[3,5,6,19]
Microglial regulation	Foxc1, IκBα, CD137L, miR-25-3p, miR-494	Early: protective Foxc1 signaling; Sustained: S100B/RAGE, IL-17A from Th1/Th17	M1/M2 balance, synaptic pruning, BBB integrity maintenance vs. disruption	[16,17,18,21,23,24]
Treg-mediated suppression	IL-10, TGF-β, AREG-EGFR, α7nAChR	Cholinergic signaling, brain infiltration via BBB	Microglial cascade antagonism, astrocytic IL-6 suppression	[33,34,35]
Neutrophil/NETosis	GSDMD, PD-L1, p-STAT3, NGAL	IL-1β/IL-6 elevation, STAT3 nuclear translocation	BBB disruption, neuronal death, microglial activation	[38,40,42]
BBB functional disturbance	Occludin ↓, Claudin-5 ↓, ZO-1 ↓, MMP-2/9 ↑, ROCK ↑, NF-κB ↑	TNF-α, IL-1β, glycocalyx shedding (syndecan-1)	Paracellular permeability ↑, early reversible leakage	[44,45,46]
BBB structural disruption	ICAM-1 ↑, VCAM-1 ↑, E-selectin ↑, caspase-3, endothelial apoptosis	Mitochondrial ATP ↓, Ca^2+^ overload, ROS-NLRP3 activation	Immune cell infiltration, microthrombosis, persistent damage	[47,48,49,50]
Autophagy/apoptosis	PINK1/Parkin, VDAC1, IRGM1, sestrin 2, ULK1, mTOR	CXCR5 ↓, p38 MAPK/NF-κB/STAT3, Nogo-A/ROS-p-SHP2	Mitochondrial quality control, neuronal survival vs. death	[59,60,63,64,115]
Pyroptosis	NLRP3, caspase-1, GSDMD, P2X7R, pannexin-1, TRIM45, Maf1	PAMPs/DAMPs, K63-ubiquitination of Atg5, TXNIP	IL-1β/IL-18 release, membrane pore formation, LDH/HMGB1 release	[75,76,77,78,79,80,81]
Ferroptosis	GPX4 ↓, SLC7A11/xCT ↓, PEBP-1/15-LOX/ACSL4 ↑, TFRC, Fpn1	Glutathione depletion, iron accumulation, lipid peroxidation	Membrane rupture, oxidative/antioxidant imbalance	[88,89,90,91,92]
Cholinergic dysfunction	ChAT ↓, AChE ↑, α7nAChR ↓, JAK2/STAT3 ↓, CAP impairment	Systemic inflammation, vagal tone reduction	Microglial activation threshold ↓, anti-inflammatory pathway failure	[7,8]
Glutamate excitotoxicity	Glx/Cr ↑, NMDAR/AMPAR-CREB-BDNF, PSD-95 ↓	TNF-α-induced glial glutamate release, astrocytic reuptake ↓	Synaptic plasticity damage, cognitive impairment	[19,93,95,97]
Gut–brain axis dysregulation	Butyrate ↓, propionate ↓, IPA ↓, SCFA-producing bacteria ↓	Dysbiosis (Bacteroides ↓, Bifidobacterium ↓), gut epithelial exosomes	Microglial JNK/NF-κB activation, tight junction downregulation	[104,105,106,107,109]

**Table 3:** Evidence base and translational barriers by pathological component.

Component	Primary Evidence Base	Key Preclinical Findings	Available Clinical Evidence	Translational Barriers	Priority Actions
Neuroinflammatory storm	CLP/LPS rodents	TNF-α/IL-1β drive microglial activation and mitochondrial damage; CAP impairment reduces anti-inflammatory capacity [3,5,6,7]	CSF cytokine elevation correlates with severity; no interventional trials [42]	Species differences in microglial phenotypes; anti-cytokine trials in sepsis failed (IL-1Ra, anti-TNF); poor BBB penetration of biologics	Microglial PET ligand development; patient stratification by CSF cytokine profile; small molecule CAP activators with CNS penetration
BBB disruption	Two-photon microscopy (rodents), MRI (humans)	Temporal progression: functional (TJ internalization) → structural (basement membrane degradation, immune infiltration) [44,47,48]	Dynamic contrast-enhanced MRI detects leakage; serum S100β/GFAP as biomarkers [88]	Temporal resolution mismatch; gadolinium contraindications in renal failure; no validated stage-specific therapies; TJ-targeted agents lack specificity	Non-gadolinium MRI contrast agents; tight junction-targeted therapeutics with imaging guidance; stage-stratified intervention trials
Multimodal cell death	CLP/LPS models, primary neuronal cultures	Pyroptosis dominates acute phase (IL-1β release); ferroptosis implicated in delayed injury (lipid peroxidation); autophagy exhibits context-dependent protection/destruction [75,88,115]	Limited post-mortem studies; no clinical cell death modality markers	Relative contribution of each modality in human SAE unknown; pan-cell death inhibitors lack specificity; GSDMD/Drp1 inhibitors lack human data	Cell death modality biomarkers (caspase-1 for pyroptosis, MDA/4-HNE for ferroptosis); stage-specific inhibition; genetic stratification
Neurotransmitter imbalance	MRS imaging (rodents), CSF metabolomics	Glx/Cr ratio correlates with cognitive impairment; cholinergic deficit reduces microglial activation threshold [7,95]	Case–control CSF studies; no prospective biomarker validation [95]	Neurotransmitter imbalance MRS imaging (rodents), CSF metabolomics [93,95] Glx/Cr ratio correlates with cognitive impairment; cholinergic deficit reduces microglial activation threshold [7,95] Case–control CSF studies; no prospective biomarker validation Heterogeneous measurement methods; lack of standardized cutoff values; rapid neurotransmitter fluctuation; receptor modulators have narrow therapeutic windows	Standardized MRS protocols; point-of-care cholinergic metabolite assays; receptor occupancy imaging; allosteric modulators with improved selectivity
Gut–brain axis	FMT, probiotic intervention in SAE models	SCFA restoration (butyrate, propionate) improves BBB and cognition; FMT modulates neurotransmitter balance [104,105,109]	Retrospective cohort studies (ammonia metabolism, nonhepatic hyperammonemia) [111,112]	Human microbiota composition differs markedly from rodents; FMT safety in immunocompromised unproven; donor variability; metabolite formulations lack standardization	Defined bacterial consortia; purified metabolite formulations (butyrate, IPA); autologous FMT after ICU discharge; pharmacokinetic studies of SCFA analogs

## Data Availability

No new data were created or analyzed in this study. All data supporting the conclusions of this review are derived from the published literature, which is cited in the reference list. The full-text articles included in this review are publicly available through PubMed, Web of Science, and Embase databases.

## Abbreviations

The following abbreviations are used in this manuscript:

5-HT	5-Hydroxytryptamine (Serotonin)
Aβ	Amyloid-β
ACh	Acetylcholine
AChE	Acetylcholinesterase
AChR	Acetylcholine Receptor
AKT	Protein Kinase B
ALFF	Amplitude of Low-Frequency Fluctuation
AMPAR	α-Amino-3-hydroxy-5-methyl-4-isoxazolepropionic Acid Receptor
AQP4	Aquaporin-4
ARE	Antioxidant Response Element
AREG	Amphiregulin
ATP	Adenosine Triphosphate
BBB	Blood–Brain Barrier
BDNF	Brain-Derived Neurotrophic Factor
BMEC	Brain Microvascular Endothelial Cell
CAP	Cholinergic Anti-inflammatory Pathway
ChAT	Choline Acetyltransferase
CLP	Cecal Ligation and Puncture
CNS	Central Nervous System
CREB	cAMP Response Element-Binding Protein
CXCR5	C-X-C Chemokine Receptor Type 5
DAMP	Damage-Associated Molecular Pattern
DHA	Dihydroartemisinin
DMT1	Divalent Metal Transporter 1
DRP1	Dynamin-Related Protein 1
EE	Environmental Enrichment
EGFR	Epidermal Growth Factor Receptor
FMT	Fecal Microbiota Transplantation
FTH1	Ferritin Heavy Chain 1
Fpn1	Ferroportin 1
Glx	Glutamate + Glutamine
GPX4	Glutathione Peroxidase 4
GSDMD	Gasdermin D
HMGB1	High Mobility Group Box 1
HO-1	Heme Oxygenase-1
HPC	Hippocampus
HtrA2	High Temperature Requirement Protein A2
ICAM-1	Intercellular Adhesion Molecule-1
IFN-γ	Interferon Gamma
IL	Interleukin
iNOS	Inducible Nitric Oxide Synthase
IPA	Indolepropionic Acid
IRGM1	Immunity-Related GTPase Family M Member 1
JAK2	Janus Kinase 2
JNK	c-Jun N-Terminal Kinase
KYNA	Kynurenic Acid
LPS	Lipopolysaccharide
MAPK	Mitogen-Activated Protein Kinase
MAR1	Maresin 1
MD2	Myeloid Differentiation Factor 2
MDA	Malondialdehyde
MFG-E8	Milk Fat Globule-Epidermal Growth Factor 8
MMP	Matrix Metalloproteinase
mPFC	Medial Prefrontal Cortex
mPTP	Mitochondrial Permeability Transition Pore
NAD+	Nicotinamide Adenine Dinucleotide
NADPH	Nicotinamide Adenine Dinucleotide Phosphate
NETs	Neutrophil Extracellular Trap
NF-κB	Nuclear Factor Kappa-Light-Chain-Enhancer of Activated B Cells
NGAL	Neutrophil Gelatinase-Associated Lipocalin
NLRP3	NOD-Like Receptor Family Pyrin Domain Containing 3
NMDAR	N-Methyl-D-Aspartate Receptor
NO	Nitric Oxide
NOX2	NADPH Oxidase 2
Nrf2	Nuclear Factor Erythroid 2-Related Factor 2
Omi	Omi/HtrA2 Serine Protease
P2X7R	Purinergic Receptor P2X 7
PAMP	Pathogen-Associated Molecular Pattern
PD-L1	Programmed Death-Ligand 1
PEBP-1	Phosphatidylethanolamine-Binding Protein 1
PG	Propylene Glycol
PGC-1α	Peroxisome Proliferator-Activated Receptor Gamma Coactivator 1-Alpha
PINK1	PTEN-Induced Putative Kinase 1
PLX5622	CSF1R Inhibitor
PS	Phosphatidylserine
PUFA	Polyunsaturated Fatty Acid
RAGE	Receptor for Advanced Glycation End-Products
ROCK	Rho-Associated Protein Kinase
ROS	Reactive Oxygen Species
SAE	Sepsis-Associated Encephalopathy
SCFA	Short-Chain Fatty Acid
Sirt1	Sirtuin 1
Sirt3	Sirtuin 3
SLC7A11	Solute Carrier Family 7 Member 11
SOD	Superoxide Dismutase
STAT3	Signal Transducer and Activator of Transcription 3
TGF-β	Transforming Growth Factor Beta
Th	T Helper Cell
TJ	Tight Junction
TLR	Toll-Like Receptor
TNF-α	Tumor Necrosis Factor Alpha
Tregs	Regulatory T Cells
TXNIP	Thioredoxin-Interacting Protein
UCP2	Uncoupling Protein 2
VCAM-1	Vascular Cell Adhesion Molecule-1
VDAC1	Voltage-Dependent Anion Channel 1
VEGF-C	Vascular Endothelial Growth Factor C
XIAP	X-Linked Inhibitor of Apoptosis Protein
α7nAChR	Alpha-7 Nicotinic Acetylcholine Receptor
γδT	Gamma-Delta T Cell
